# Comorbidities, repeated hospitalizations, and age ≥ 80 years as indicators of anemia development in the older population

**DOI:** 10.1007/s00277-018-3321-x

**Published:** 2018-04-09

**Authors:** Sylwia Sulimiera Michalak, Joanna Rupa-Matysek, Lidia Gil

**Affiliations:** 1 Faculty of Medicine and Health Sciences, University of Zielona Góra, Zielona Góra, Poland; 2 Department of Hematology and Bone Marrow Transplantation, Poznan University of Medical Sciences, Szamarzewskiego 84, 60-569, Poznań, Poland

**Keywords:** Anemia, Elderly, Comorbidities, Risk factor, Survival

## Abstract

Anemia represents a common condition among the elderly; however, its prevalence and causes are not well known. This retrospective analysis was performed on 981 patients aged ≥ 60 in Poland over 2013–2014. The prevalence of anemia was 17.2% and increased with age. The predominant causes of anemia were the following: anemia of chronic disease (33.1%), unexplained anemia (28.4%), deficiency anemia (22.5%, including iron deficiency 13%), and chemo-/radiotherapy-induced anemia (8.9%). In the multivariate logistic regression model, factors increasing the risk of anemia were the following: age ≥ 80 years (OR 2.29; 95%CI 1.19–4.42; *P* = 0.013), the number of comorbidities (two diseases OR 2.85; 95%CI 1.12–7.30; *P* = 0.029, three diseases OR 6.28; 95%CI 2.22–17.76; *P* = 0.001, four diseases OR 4.64; 95%CI 1.27–17.01; *P* = 0.021), and hospitalizations (OR 1.34; 95%CI 1.13–1.58; *P* = 0.001). After a 2-year follow-up, the cumulative survival among patients without anemia in relation to the group with anemia was 90.76 vs. 78.08% (*P* < 0.001). In the multivariate model, anemia (HR 3.33, 95%CI 1.43–7.74, *P* = 0.005), heart failure (HR 2.94, 95%CI 1.33–6.50, *P* = 0.008), and cancer (HR 3.31, 95%CI 1.47–7.49, *P* < 0.004) were all significantly correlated with mortality. In patients ≥ 60 years, the incidence of anemia increases with age, number of comorbidities, and frequency of hospitalizations and has an adverse impact on survival.

## Introduction

The population of people older than 60 years is increasing by 3.26% annually, and it has been estimated that by 2050, it will constitute 25% of the entire population [1]. High frequency of comorbidities, polypharmacotherapy, and hospitalization is commonly seen in this group of people [2–4]. Anemia is a common condition among the elderly and is increasing with age. The etiology of anemia in the elderly is often complex, and only a few studies have been performed to evaluate the impact of multiple factors, including comorbidities and hospitalizations, on the development of anemia [5–7].

The aim of the study was to evaluate the prevalence, severity, and etiology of anemia with a risk factor analysis for its development in the population aged ≥ 60 years, with respect to comorbidities and hospitalizations.

## Materials and methods

Retrospective analysis was performed on all consecutive white patients aged 60 years or over, who were under the care of a specific primary medical care clinic in Poland during 2013–2014. Primary medical clinic provided only ambulatory, outpatient care. In the case of hospitalization, the patient stayed under the care of a hospital specialist and after discharge returned to the primary medical care clinic. All patients’ data, including the level of hemoglobin and types of anemia, common comorbidities, and hospitalizations were derived from the medical records of the clinic and patient’s files. Data on all-cause mortality between the first of January 2013 and the 31st of December 2014 were based on the reports of the National Health Fund.

Both, patients aged ≥ 60 years with anemia detected in the years 2013–2014 and patients with anemia diagnosed earlier (since 2001) but aged 60 or over at the time of diagnosis, were included in the study. The incidence of anemia, comorbidities, and hospitalizations since the age of 60 were analyzed both in the entire population and also separately in the patients with and without anemia.

### Definitions of anemia and types of anemia

Anemia was defined as hemoglobin levels lower than 12 g/dl in women and 13 g/dl in men according to WHO criteria [8]. If a single morphological result with a reduced hemoglobin concentration was found in the medical records, which was not confirmed in subsequent studies and without clinical data indicating anemia, no anemia was diagnosed. Finding the presence of anemia at the age of 60 and over resulted in the patient being classified with anemia. In anemic patients, the result of morphology at the time of diagnosis of anemia was analyzed, and in patients without anemia, the last morphology result performed in the years 2012–2014. The severity of anemia was established according to the most commonly used classification subtypes: mild anemia: 10–12 g/dl in woman and 10–13 g/dl in men, moderate anemia 8–9.9 g/dl, severe anemia 6.5–7.9 g/dl, and very severe anemia under 6.5 g/dl [9]. The severity of anemia was established based on the hemoglobin concentration at the time of diagnosis.

The following types of anemia were determined: deficiency anemia’s (iron deficiency, vitamin B12 deficiency, folate acid deficiency, and complex deficiency anemia), hemorrhagic anemia, anemia of chronic disease, renal insufficiency anemia, chemo- and/or radiotherapy-induced anemia, and other types in the course of chronic liver disease, alcoholic disease, thyroid gland disease, and unexplained anemia. The definitions of each types of anemia are listed in Table 1. If a patient underwent chemo- or radiotherapy and had anemia lasting at least a month and there was a cause-and-effect relationship between the onset of anemia and cancer therapy, we classified the patient as chemo- and/or radiotherapy-induced anemia. The cause-and-effect relationship was defined as follows: before cancer therapy, there was no anemia nor any other risk factors (e.g., bleeding, inflammation markers indicative of another cause of anemia), anemia occurred during oncological treatment and lasted at least 1 month after treatment. GFR < 30 ml/min/1.73 m^2^ was considered a criterion for diagnosing anemia in the course of kidney disease [10–14].

**Table 1:** The definitions of the different types of anemia, adapted based on published data [12, 15–17]

Anemia etiology	Criteria	Comments
Iron deficiency	Serum ferritin < 30 ng/ml, TfS < 16%	Excluded if not corrected by 2 months of oral or intravenous iron therapy
Vitamin B12 deficiency	Vitamin B12 < 197 pg/ml	Excluded if not corrected by 3 months of vit. B12 therapy
Folate deficiency	Folate level < lower limit of normal	Excluded if not corrected by 3 months of folate therapy
Anemia of chronic disease	Elevations of CRP, ESR, serum ferritin > 100 ng/ml The presence of chronic or acute infection; autoimmune diseases (systemic lupus erythematosus, rheumatoid arthritis, inflammatory bowel disease)	Elevations of CRP, ESR, serum ferritin 30–100 ng/ml, considered as coexistence of iron deficiency but was eligible for ACD
Renal insufficiency anemia	eGFR < 30 ml/m^2^/min/1.73 m^2^	Normal values of CRP and ESR
Chemo- and/or radiotherapy-induced anemia	Confirmed cause-and-effect relationship with chemo-/radiotherapy	Anemia occurred during oncological treatment and lasted at least 1 month after treatment
Anemia in the course of thyroid disease	TSH < 0.1 mU/l or > 10 mU/l	Regression of anemia after normalization of thyroid dysfunction
Hemorrhagic anemia	The presence of overt bleeding with drop in hemoglobin level	
Anemia in the course of chronic liver disease	The presence of chronic liver disease: cirrhosis, chronic viral hepatitis	Other causes of anemia excluded
Alcohol-induced anemia	The presence of alcoholic illness	Other causes of anemia excluded

*CRP*, C reactive protein; *ESR*, erythrocyte sedimentation rate; *TfS*, transferrin saturation; *eGFR*, estimated Glomerular filtration rate; *TSH*, thyrotropin; *ACD* anemia of chronic disease

Anemia was defined as unexplained if, based on available patients’ records and laboratory tests, no other types of anemia were identified.

The factors that increase the likelihood of anemia were examined using the logistic regression model. The probability is expressed by the odds ratio (OR). The following variables were analyzed: gender, age, renal function (determined on the basis of eGFR), selected comorbidities (coronary heart disease, diabetes, insulin-dependent diabetes, chronic kidney disease, heart failure, chronic obstructive pulmonary disease, thyroid diseases, cancer, and chronic liver diseases), number of comorbidities, number of hospitalizations, and selected drugs (anticoagulants and aspirin).

### Demographic variables, comorbidities, hospitalizations, and procedures

Based on medical records, data on age (divided into groups 60–69, 70–79, ≥ 80), gender, hemoglobin concentration, and type of anemia was collected. Data on the incidence of common comorbidities including hypertension, coronary heart disease/cardiovascular disease, heart failure, atrial fibrillation, venous thromboembolism, diabetes including diseases with chronic complications (macroangiopathy, microangiopathy, neuropathy, and retinopathy), chronic lung disease including asthma and chronic obstructive pulmonary disease, chronic kidney disease, cancer, hematological malignancies, chronic liver diseases, rheumatic disease, and thyroid diseases were obtained. All comorbidities were diagnosed according to the most current guidelines of the relevant international societies [15–25], and results from specialist consultations and hospital discharge cards. Heart failure was diagnosed on the basis of subjective and objective symptoms as well as features of systolic or diastolic heart dysfunction in echocardiography [15]. Cancer was defined as malignant neoplasm, confirmed by histopathology, imaging and laboratory tests [25], and required treatment (active cancer). Melanoma and other skin cancers were also evaluated in the study. The appropriate treatment recorded in the documentation confirmed the diagnosis of specific diseases. The data on the use of prophylactic aspirin (75–150 mg), clopidogrel, vitamin K antagonists (VKA), and DOACs (Direct Oral Anticoagulants) were also collected.

The incidence of hospitalizations, excluding hospitalization for surgery, rehabilitation, and cancer treatment was also analyzed. In the course of hospitalization, the incidence of coronary angiography and percutaneous transluminal coronary angioplasty (PTCA) was assessed in patients with and without anemia.

The Bioethical Committee of Poznan University of Medical Sciences approved the study.

### Statistical analysis

The results are presented using methods of descriptive statistics such as the frequency (*n*), mean/medians, standard error (SE), and range. The Shapiro-Wilk test was performed to assess normality. To compare differences between the groups, the chi-squared test or the Fisher-Freeman-Halton exact test was used for categorical variables and the Mann-Whitney *U* test for continuous variables. The differences in the proportions between the groups (anemia/no anemia) were compared using the test for proportions.

Univariate logistic regression was used to evaluate potential risk factors for a higher prevalence of anemia. A multivariate analysis was performed with selected variables that were significant in the univariate analysis. In each model, the OR for each independent variable was determined with a confidence interval (CI) of 95%.

The probabilities of survival were estimated via the Kaplan-Meier method, and univariate comparisons were performed using the log-rank test. The Cox proportional hazards model was fitted to estimate the effect of the analyzed factors on the mortality. In this model, the hazard ratio (HR) for each independent variable was determined with a CI of 95%. A *P* value below 0.05 was regarded as statistically significant.

The statistical analyses were performed with STATISTICA 10 and STATISTICA Medical Package 2.0 (Stat Soft, Inc. 2012 software, Tulsa, USA).

## Results

**Fig. 1:**
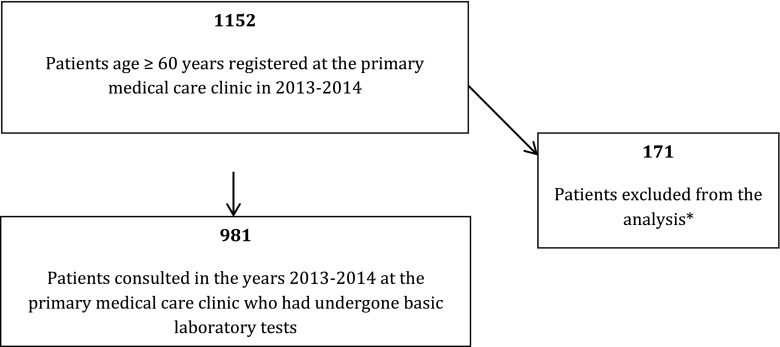
Patients in the study. *The exclusions concerned patients who were not consulted in the primary medical care clinic in 2013–2014 or who did not undergo basic laboratory tests

Of 3500 patients under the care of one primary medical care clinic, 1152 were 60 years old or older, of this group 981 patients were included into the study (Fig. 1). Detailed data concerning the demographic characteristics of the studied population is shown in Table 2.

**Table 2:** Demographic characteristics of studied population

Characteristic	Result
Total population, *n* Median age (range), years	981 68 (60–99)
60–69 years, *n* (%)	544 (55.5)
70–79 years, *n* (%)	274 (27.9)
≥ 80 years, *n* (%) Gender: female/male *n* (%)	163 (16.6) 594(60.6)/387(39.4)

In the analyzed population, the mean hemoglobin concentration in women was lower than in men (13.15 vs. 13.95 g/dl; *P* < 0.001). Anemia was found in 169 patients (17.2%), including 90 women (9.2%) and 79 men (8.0%). The prevalence of anemia was higher in men than women (20.4 vs. 15.2%; *P* = 0.038). Anemia incidence increased statistically significantly with age groups (60–69 vs. 70–79 *P* < 0.001, 60–69 vs. 80 or more *P* < 0.001, 70–79 vs. 80 or more *P* < 0.001) (Table 3).

**Table 3:** Prevalence of anemia according to age groups (60–69, 70–79, 80 and over) and gender

Patients with anemia	60–69 years	70–79 years	≥ 80 years	*P* value		
	*n* (%)			60–69 vs. 70–79	60–69 vs. ≥ 80	70–79 vs. ≥ 80
Total	56 (10.3)	55 (20.1)	58 (35.6)	< 0.001	< 0.001	< 0.001
Women	21 (6.9)	31 (18.0)	38 (32.8)	< 0.001	< 0.001	< 0.001
Men	35 (14.7)	24 (23.5)	20 (42.6)	0.225	0.015	0.238

*P* < 0.05—statistically significant

The anemia was mild in 118 patients (69.8%), moderate in 40 patients (23.7%), severe in 6 patients (3.6%), and very severe in 5 patients (3.0%). The group of men aged 80 or over was more likely to have severe anemia than younger ones (*P* = 0.030).

### Types of anemia

**Fig. 2:**
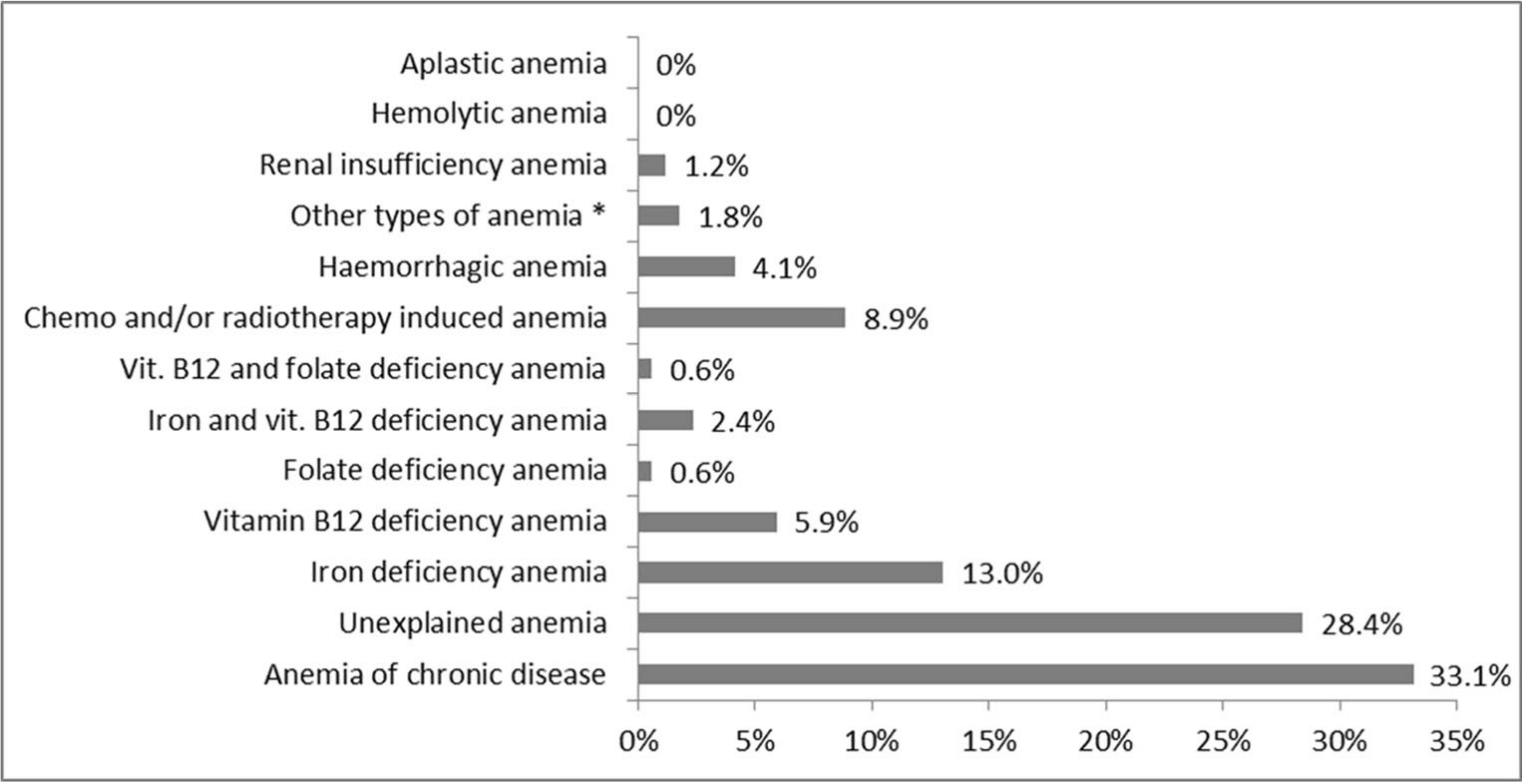
Types of anemia in the studied population aged ≥ 60 years. *Other types of anemia included the following: anemia in the course of chronic liver disease (*n* = 2) and alcohol induced anemia (*n* = 1)

The predominant causes of anemia were the following: anemia of chronic disease (33.1%), unexplained anemia (28.4%), and deficiency anemia (22.5%, including iron deficiency constituting 13% of all cases), and 8.9% of anemia resulted from cancer treatment (chemo-/radiotherapy-induced anemia). The incidence of other types of anemia in the studied population is presented in Fig 2. No case of aplastic anemia or hemolytic anemia was diagnosed during observation.

The analysis of the prevalence of anemia in different age groups showed a more significant increase in the incidence of anemia of chronic disease (ACD) in the 60- to 69-year group than in the 70- to 79-year group (21.4 vs. 41.8%, *P* = 0.021). Similarly, the incidence of chemo-/radiotherapy-induced anemia in the 60- to 69-year group was higher compared to the 70- to 79-year group (19.6 vs. 5.5%, *P* = 0.024) and the ≥ 80 group (19.6 vs. 1.7% *P* = 0.002).

### Association of anemia with age, comorbidities, and hospitalization

In comparison to the patients without anemia, those with anemia were older (median age 75 vs. 67, *P* < 0.001), had a higher prevalence of comorbidities (median 3 vs. 2, *P* < 0.001), and statistically significantly more often had cardiac complications, venous thromboembolism, diabetes including diseases with chronic complications, chronic obstructive pulmonary disease, thyroid diseases, chronic kidney disease, cancer, chronic liver diseases, or rheumatic disease. The detailed clinical characteristics of the patients with or without anemia are provided in Table 4.

**Table 4:** Clinical characteristics of the patients with or without anemia

Characteristics	Total population *n* = 981	Patients with anemia *n* = 169	Patients without anemia *n* = 812	*P* value
Median age, years	68	75	67	< 0.001
Number of comorbidities, median (range)	2 (0–12)	3 (0–9)	2 (0–8)	< 0.001
Comorbidities, *n* (%)				
Hypertension	772 (78.7)	146 (86.4)	626 (77.1)	0.007
Coronary heart disease	319 (32.5)	76 (45.0)	243 (29.9)	< 0.001
Heart failure	106 (10.8)	43 (25.4)	63 (7.8)	< 0.001
Atrial fibrillation	102 (10.4)	35 (20.7)	67 (8.3)	< 0.001
Venous thromboembolism	34 (3.5)	14 (8.3)	24 (3.0)	0.001
Diabetes	245 (25.0)	53 (31.4)	192 (23.6)	0.035
Diabetes with complications	143(14.6)	40 (23.7)	103 (12.7)	< 0.001
Thyroid diseases	182 (18.6)	42 (24.9)	140 (17.2)	0.021
Pulmonary disease	118 (12.0)	34 (20.1)	84 (10.3)	< 0.001
Asthma	51 (5.2)	8 (4.7)	43 (5.3)	0.765
COPD	39 (4.0)	12 (7.1)	27 (3.3)	0.022
Chronic kidney disease	102 (10.4)	49 (29.0)	53 (6.5)	< 0.001
Cancer	101 (10.3)	44 (26.0)	57 (7.0)	< 0.001
Hematological malignancies	7 (0.7)	6 (3.6)	1 (0.1)	< 0.001
Chronic liver diseases	33 (3.4)	19 (11.2)	14 (1.7)	< 0.001
Rheumatic diseases	11 (1.1)	7 (4.1)	4 (0.5)	< 0.001
Drugs, *n* (%)				
Aspirin	259 (26.4)	82 (48.5)	177 (21.8)	< 0.001
Clopidogrel	68 (6.9)	27 (15.9)	41 (5)	< 0.001
VKA	78 (8)	30 (17.8)	48 (5.9)	< 0.001
DOACs	25 (2.5)	10 (5.9)	15 (1.8)	0.002
Number of hospitalizations, median (range)	0 (0–12)	2 (0–12)	0 (0–11)	< 0.001
Coronary angiography, *n* (%)	140 (14.3)	53 (31.4)	87 (10.7)	< 0.001
PTCA, *n* (%)	84 (8.6)	35 (20.7)	49 (6.0)	< 0.001

*COPD*, chronic obstructive pulmonary disease; *VKA*, vitamin K antagonists; *DOACs* direct oral anticoagulants; *PTCA* percutaneous transluminal coronary angioplasty

*P* < 0.05—statistically significant

The analysis of the prevalence of comorbidities in the age groups 60–69, 70–79, and ≥ 80 showed that heart failure was significantly more common in patients with anemia (*P* = 0.002, *P* = 0.018, and *P* = 0.013, respectively), chronic kidney disease (*P* = 0.013, *P* < 0.001, *P* = 0.013, respectively), and chronic liver disease (*P* < 0.001, *P* = 0.013, *P* = 0.007, respectively). Patients with anemia were more often hospitalized (median 2 vs. 0, *P* < 0.001) and more often had coronary angiography and/or PTCA than those without anemia (*P* < 0.001).

### Risk factors for the anemia development

From the risk factors analyzed for the development of anemia (age, gender, kidney function based on eGFR, comorbidities, and hospitalizations), most of them were statistically significant based on the univariate logistic regression model (Table 5). In the multivariate logistic regression model, age ≥ 80 years (OR 2.29; 95%CI 1.19–4.42; *P* = 0.013), the number of comorbidities (two diseases OR 2.85; 95%CI 1.11–7.29; *P* = 0.029, three diseases (OR 6.28; 95%CI 2.22–17.76; *P* = 0.001), four diseases OR 4.64; 95%CI 1.26–17.00; *P* = 0.021), and number of hospitalizations (OR 1.34; 95%CI 1.13–1.58; *P* = 0.001) were all significantly associated with a higher prevalence of anemia (Table 6).

**Table 5:** Univariate regression analysis for occurrence of anemia

Parameter	OR	95%CI	P value
Sex (male)	1.44	1.03–2.01	0.033
Age, year			
70–79	2.19	1.46–3.28	< 0.001
≥ 80	4.81	3.15–7.35	< 0.001
eGFR (ml/min/1.73 m^2^)			
< 15	1.00	–	–
15–29	7.85	2.01–30.61	0.003
30–59	3.44	2.08–5.68	< 0.001
60–89	0.99	0.64–1.55	0.969
Coronary heart disease	1.91	1.36–2.68	< 0.001
Diabetes	1.48	1.03–2.12	0.036
Insulin-treated diabetes	2.67	1.53–4.65	0.001
Chronic kidney disease	5.99	3.85–9.32	< 0.001
Heart failure	4.06	2.64–6.24	< 0.001
COPD	2.22	1.1–4.48	0.026
Thyroid diseases	1.59	1.07–2.35	0.021
Cancer	4.66	3.01–7.21	< 0.001
Chronic liver diseases	7.22	3.54–14.72	< 0.001
Drugs			
Anticoagulants (VKA, DOACs)	3.58	2.28–5.61	< 0.001
Antiplatelet agents (Aspirin)	3.38	2.4–4.77	< 0.001
Number of comorbidities			
1	2.14	1.26–3.65	0.005
2	4.38	2.56–7.49	< 0.001
3	10.92	6.13–19.47	< 0.001
4	13.22	5.95–29.37	< 0.001
5	14.04	2.69–73.33	0.002
Hospitalization	1.46	1.28–1.67	< 0.001

*OR*, odds ratio; *CI*, confidence interval; *COPD*, chronic obstructive pulmonary disease; *VKA*, vitamin K antagonists; *DOACs* direct oral anticoagulants

*P* < 0.05—statistically significant

**Table 6:** Multivariate regression analysis for occurrence of anemia

Parameter	OR	95%CI	P value
Age, years			
70–79	1.35	0.73–2.52	0.338
≥ 80	2.29	1.19–4.42	0.013
Number of comorbidities			
1	1.42	0.57–3.53	0.454
2	2.85	1.12–7.30	0.029
3	6.28	2.22–17.76	0.001
4	4.64	1.26–17.01	0.021
5	1.37	0.13–14.52	0.793
Hospitalization	1.34	1.13–1.58	0.001

*OR*, odds ratio; *CI*, confidence interval

*P* < 0.05—statistically significant

### All-cause mortality

Of the 981 patients, 40 patients died (4.1%) during the 2-year follow-up, including 21 (12.4%) patients with anemia and 19 (2.34%) in the group without anemia.

**Fig. 3:**
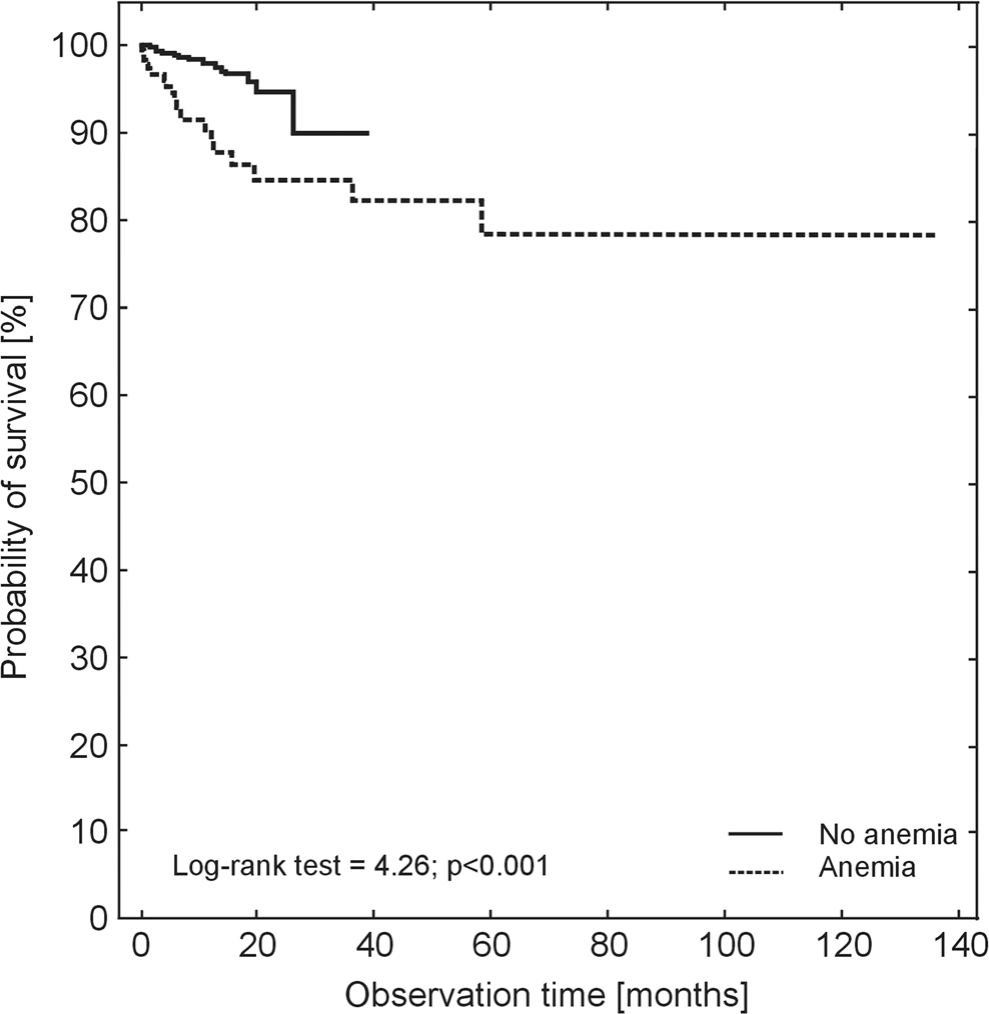
Cumulative survival probability of the studied patients aged ≥ 60 years with or without anemia

After a 1-year follow-up, the cumulative survival among patients with anemia in relation to the group without anemia was 87.63 vs. 97.54% and at the end of the study 78.08% (SE ± 0.055) and 90.76% (SE ± 0.049) respectively, and the difference was statistically significant (*P* < 0.001), Fig 3.

To determine factors influencing survival, the variables including age, gender, selected comorbidities, hospitalizations, and also the presence and types of anemia were analyzed. In the univariate analysis, factors that significantly increased the risk of death were the following: age ≥ 80 years (HR 7.6, 95%CI 3.47–16.65, *P* < 0.001), anemia (HR 4.32; 95%CI 2.18–8.56, *P* < 0.001, heart failure (HR 5.01, 95%CI 2.56–9.80, *P* < 0.001), and cancer (HR 4.82, 95%CI 2.44–9.54, *P* < 0.001) (Table 7).

**Table 7:** Univariate analyses determining factors affecting mortality in the population aged 60 or over

Parameter/variables	HR	95%CI	*P* value
Male gender	1.03	0.53–2.01	0.921
Age, years			
70–79	1.28	0.46–3.61	0.635
≥ 80	7.60	3.47–16.65	< 0.001
eGFR vs. > 90 ml/min/1.73 m^2^			
< 15	1.99E-19	–	–
15–29	1.08E-17	–	–
30–59	0.88	0.29–2.65	0.811
60–89	0.95	0.42–2.18	0.908
Diabetes	0.60	0.25–1.44	0.252
Diabetes with complications	0.85	0.33–2.20	0.740
Heart failure	5.01	2.56–9.8	< 0.001
Cancer	4.82	2.44–9.54	< 0.001
Anemia	4.32	2.18–8.56	< 0.001
Acute coronary syndrome	1.75	0.76–4.03	0.184
Stroke	1.28	0.39–4.20	0.679
Types of anemia			
Anemia of chronic disease	1.54	0.63–3.77	0.350
Unexplained anemia	0.87	0.32–2.39	0.785
Iron deficiency anemia	0.28	0.04–2.06	0.209
Vitamin B12 deficiency anemia	0.82	0.19–3.57	0.795
Folate deficiency anemia	6.14E-16	–	1.000
Chemo- and/or radiotherapy-induced anemia	1.89	0.55–6.46	0.312
Hemorrhagic anemia	1.14	0.15–8.53	0.901
Renal insufficiency anemia	6.21E-16	–	1.000
Other types of anemia	2.18E-16	–	1.000

*HR*, hazard ratio; *CI*, confidence interval

*P* < 0.05—statistically significant

In the multivariate model for the whole studied population, three factors including anemia (HR 3.33; 95%CI 1.43–7.74; *P* = 0.005), cancer (HR 3.31; 95%CI 1.47–7.49; *P* = 0.004), and heart failure (HR 2.94; 95%CI 1.33–6.50; *P* = 0.008) all significantly correlated with the risk of mortality (Table 8).

**Table 8:** Multivariate analysis determining factors affecting mortality in the population aged 60 or over

Parameter	HR	95%CI	*P* value
Anemia	3.33	1.43–7.74	0.005
Heart failure	2.94	1.33–6.51	0.008
Cancer	3.31	1.47–7.49	0.004

*HR*, hazard ratio; *CI* confidence interval

*P* < 0.05—statistically significant

## Discussion

In the studied population aged 60 years or over, anemia as defined according to WHO criteria was found in 17.2% of patients, including 9.2% of women and 8.0% of men. Our results are in line with the observations of other authors concerning the prevalence of anemia in the elderly: 9.6% in the USA [26], 14.2% in Italian population [27], 17.4% in Poland [28], 13.6% in the Korean population [29], and 21% among Austrian people [30]. Based on the meta-analysis of patients aged 60 or over, it was estimated that the prevalence of anemia in men ranges from 2.9 to 61%, while in women from 3.3 to 41% [31].

Consistent with literature on the elderly population [10, 32, 33], our data show that three types of anemia were predominant: ACD (33.1%), unexplained anemia (28.4%), and deficiency anemia (22.5%) with iron deficiency reaching 13% of all anemia cases. The high prevalence of unexplained anemia needs attention—it has been reported by many authors, as affecting 16–40% of elderly patients with anemia [10, 12, 30, 32, 34]. It is believed that some unexplained anemia cases are actually undiagnosed myelodysplastic syndrome (MDS), especially because the probability of MDS increases with age and initially, apart from anemia, there may be no other hematological abnormalities. Based on previous reports, MDS occurs in 5 to 15% of patients with anemia [10, 30, 32, 35], but other hematological malignancies may also be the case [12]. The high rate of unexplained anemia indicates the necessity for detailed hematologic diagnostics and also further research on anemia in the elderly. The development and greater availability of molecular tests may hopefully improve the diagnosis of unexplained anemia and other cytopenia. The presence of two or more somatic mutations in the genes (mutations that repeat in hematological malignancies) is associated with a higher probability of developing clonal disease [36]. Unfortunately, for the time being, these methods do not provide an answer, in a large percentage of patients, as to the cause of the anemia.

Our data revealed a significant proportion (8.9%) of anemia associated with cancer treatment (chemo-/radiotherapy-induced anemia). This is also an important issue, because anemia is an independent risk factor for cancer mortality [37], and its successful treatment improves the quality of life and outcomes of cancer patients [38]. It has been shown in our study that anemia associated with cancer therapy is more likely to occur at 60–69 years of age compared to 70–79 years and 80 years or more, which seems surprising, since the incidence of cancer increases with age, as well as the likelihood of anemia in the course of cancer and its treatment [39, 40].

The prevalence of anemia of chronic disease in our analysis was more common in the 70- to 79-year group than in the age group of 60 to 69, which is supported by observations of the increased incidence with age disorders associated with increased proinflammatory activity [41]. The prevalence of other types of anemia in subsequent age groups (60–69 years, 70–79 years, and 80 years and over) was similar.

Patients with anemia were older, had a higher prevalence of comorbidities, more often used antiplatelet agents and oral anticoagulants, and were more often hospitalized. On the other hand, in the multivariate model, the increased number of comorbidities (2 to 4) significantly increased the risk of anemia development in the studied population. Comorbidities were more frequently found in patients with anemia, which has been also observed in other studies [5, 10, 42, 43]. Although a lot of research indicates the association between anemia and heart failure and chronic kidney disease, the link between anemia and chronic liver disease has not been highlighted. In the present study, a higher incidence of anemia in patients with chronic liver disease was demonstrated. The pathogenesis of anemia in the course of chronic liver disease is complex and is associated with acute and chronic gastrointestinal blood loss due to esophageal varices and portal gastropathy, folate deficiency, hipersplenism, direct toxicity due to alcohol, anemia of chronic disease, hemolysis, and hemostatic abnormalities in liver failure. In cirrhosis, especially that caused by alcohol abuse, folate deficiency, and vitamin B12 deficiency may be found [44]. Anemia in the course of chronic liver disease is an example of a phenomenon often seen in elderly patients, a common etiology of anemia but with a complex pathogenesis comprising various types of anemia. Similarly, complex pathogenesis of anemia occurs in chronic kidney disease, alcoholism, endocrine disorders, and chemo-/radiotherapy-associated anemia.

In our study, patients aged 60 or more with anemia were more frequently hospitalized than those without anemia. Thus, the patients with anemia were older, which could have had an impact on the number of comorbidities and need for hospitalization. However, regardless of age, all patients with anemia more often had significantly chronic liver disease, heart failure, and chronic kidney disease.

Our observation is in line with other studies, which also reported that patients with anemia were older than those without anemia [5, 43], and this is consistent with an increased incidence of anemia with age. The association of anemia with hospitalizations has also been demonstrated in other studies [5, 43, 45]. Based on a 4-year observation of the Established Populations for Epidemiologic Studies of the Elderly (EPESE), it has been shown that anemia is related to a significant increase in hospitalizations and prolonged hospital stays [5]. The occurrence of hospital-acquired anemia (HAA) may also be associated with invasive medical procedures, excessive testing, and modern therapy, especially using thrombolytic, antithrombotic, and antiplatelet agents. Depending on the study population and reasons for hospitalization (e.g., acute coronary syndrome, kidney disease), it is estimated that the prevalence of HAA varies from 25 to 74% [46]. In the course of hospitalization, patients with anemia were more likely to undergo medical procedures such as coronary angiography and PTCA. The relationship between anemia and these procedures is complex. The procedures themselves promote the occurrence of anemia, as was observed in one out of every three patients with acute coronary syndrome, 1 month after discharge from hospital [47]. Possibly, one of the reasons that the patients required hospitalization and the implementation of these procedures was the presence of anemia and its impact on the cardiovascular system. It appears that the problem of hospital-acquired anemia in elderly patients has thus far been insufficiently observed.

In our study, the relation between comorbidities, number of hospitalizations, and anemia has been confirmed in the logistic regression analyses. In the univariate analysis, patients with a coexistence of two to five comorbidities have a nearly 2 to 14-fold increased risk of anemia development. In the multivariate logistic regression model, factors increasing the risk of anemia were age ≥ 80 years, the number of comorbidities (from two to four diseases) and hospitalizations. Due to the small number of patients with five concomitant diseases and anemia, we failed to show any relationship between the coexistence of five diseases and anemia development. To our knowledge, only a few studies have investigated comorbidities and hospitalizations as factors for anemia development, and no study has highlighted the increased risk of anemia with the number of comorbidities and the number of hospitalizations. In a multivariate logistic regression analysis, only Penninx et al. reported the following factors significantly associated with the higher prevalence of anemia: older age, black race, cancer, kidney disease, hospitalization during the previous year, and BMI [5]. There seems to be reciprocal feedback: the presence of comorbidities or the treatment used promotes the onset of anemia [48–53]. Anemia exacerbates the course of a number of diseases [51, 54–56] and may be one of the factors that contribute to the need to perform certain medical procedures (e.g., coronary angiography) or hospitalization (especially due to heart failure) [42, 54, 57], and both procedures and hospitalizations may be conducive to the development of anemia [47, 58].

In the present study, anemia at age ≥ 60 years had a negative impact on survival, and was, in addition to heart failure and cancer, one of the most important risk factors for death. This is in agreement with data from previous studies that have also shown the negative effect of anemia on survival [5, 43, 45, 59]. Similarly, Zakai et al. have shown that anemia is an important risk factor for mortality, not only in relation to cardiovascular disease, heart failure, and cancer, but also diabetes [59].

The present study was conducted on a large, well-defined group of patients in advanced age and included analysis of many factors affecting their health condition. The conclusion can be drawn that anemia is common among the studied population and its prevalence increases with age. The presence of anemia increases the risk of death, and additionally, there is an association between anemia and comorbities and hospitalizations. Based on our results, it is possible to identify the group of elderly patients at high risk of the development of anemia, which is made up of people aged 80 and over, with two or more comorbidities, and those who require hospitalization. Identification of the risk group may provide the basis for recommendations for more frequent blood morphology testing in the population at risk of anemia development.

For two decades, there has been no change in the situation of elderly patients with unexplained anemia and therefore no effective treatment is possible. The diagnosis of unexplained anemia in nearly one third of patients indicates, on the one hand, the need for a more in-depth diagnosis, and on the other hand, further research to understand the causes of anemia in this age group.
